# The Evolving Role of Next-Generation Sequencing in Screening and Diagnosis of Hemoglobinopathies

**DOI:** 10.3389/fphys.2021.686689

**Published:** 2021-07-27

**Authors:** Ahlem Achour, Tamara T. Koopmann, Frank Baas, Cornelis L. Harteveld

**Affiliations:** ^1^Department of Clinical Genetics/LDGA, Leiden University Medical Center, Leiden, Netherlands; ^2^Department of Congenital and Hereditary Diseases, Charles Nicolle Hospital, Tunis, Tunisia

**Keywords:** NGS, WES, WGS, β-thalassemia, sickle cell disease, α-thalassemia, hemoglobinopathy diagnostics

## Abstract

During the last few years, next-generation sequencing (NGS) has undergone a rapid transition from a research setting to a clinical application, becoming the method of choice in many clinical genetics laboratories for the detection of disease-causing variants in a variety of genetic diseases involving multiple genes. The hemoglobinopathies are the most frequently found Mendelian inherited monogenic disease worldwide and are composed of a complex group of disorders frequently involving the inheritance of more than one abnormal gene. This review aims to present the role of NGS in both screening and pre- and post-natal diagnostics of the hemoglobinopathies, and the added value of NGS is discussed based on the results described in the literature. Overall, NGS has an added value in large-scale high throughput carrier screening and in the complex cases for which common molecular techniques have some inadequacies. It is proven that the majority of thalassemia cases and Hb variants can be diagnosed using routine analysis involving a combined approach of hematology, hemoglobin separation, and classical DNA methods; however, we conclude that NGS can be a useful addition to the existing methods in the diagnosis of these disorders.

## Introduction

Hemoglobinopathies are a heterogeneous group of disorders comprising the most common recessive diseases encountered worldwide and are posing a major health problem. Therefore, they comprise one of the most studied examples of Mendelian inherited monogenic diseases. Diseases resulting from Mendelian inheritance are caused by single-gene mutations and have an overall estimated frequency of 40–82 per 1,000 live births (Christianson and Howson, 2006; Weatherall and Clegg, 1996; Weatherall, 2010; Piel et al., 2013). The hemoglobinopathies are complex since frequently more than one type of hemoglobinopathy is inherited simultaneously. DNA variants of the globin genes may cause changes in the globin structure leading to the production of abnormal hemoglobin, while the variants affecting the gene expression result in reduced production of a globin chain (of normal structure) resulting in thalassemia. Approximately 7% of the world population is a healthy carrier of hemoglobinopathy (World Health Organization [WHO], 1987). Inheritance of some combinations of mutations gives rise to severe diseases, notably sickle cell disease and beta-thalassemia major, and causes major health problems. It is estimated that approximately 350,000 newborns (annually) are found to have either of these conditions. Although the prognosis for hemoglobinopathies has been markedly improved, in general, lifelong treatment is required. Because of the high prevalence, many countries have implemented national screening programs to detect carriers and to offer counseling to couples at risk to reduce the number of affected births (Giordano, 2009; Michlitsch et al., 2009; Lobitz et al., 2018). In the vast majority of cases, hemoglobinopathies are caused by point mutations or large deletions involving the globin genes. Occasionally, mutations in transacting genes and intergenic regions have been reported (Thein, 2013; Farashi and Harteveld, 2018). Understanding the different molecular mechanisms leading to hemoglobinopathies is important to provide an adequate molecular diagnosis. According to the EMQN best practice policy for molecular and hematology methods for carrier identification and prenatal diagnosis of the hemoglobinopathies (Traeger-Synodinos et al., 2015), the red cell hematology is followed by biochemical assays by using high pressure liquid chromatography (HPLC), isoelectric focusing (IEF), and/or capillary electrophoresis (CE) and confirmation at the DNA level. Based on hematology and biochemical results, molecular analysis is performed using Gap-PCR for the identification of DNA deletions or gene rearrangements, direct sequencing analysis, and/or multiplex ligation-dependent probe amplification (MLPA) (Traeger-Synodinos et al., 2015; Harteveld, 2018). The sequential workflow in which only one gene is investigated at a time by Sanger sequencing may be time-consuming and costly and may miss some rare causative variants, resulting in a delay in genetic counseling and unresolved cases. In addition, current screening methods may miss causative alpha mutations giving rise to severe hemoglobin H (HbH) disease (He et al., 2017). Currently, diagnosis of an increasing number of genetic diseases is performed by large-scale parallel sequencing of disease gene panels instead of a sequential gene by gene Sanger sequencing, and targeted panels or whole exome sequencing (WES) are used to increase the speed of diagnosis and reduce cost in many genetic diseases.

This review aims to present the different applications of next-generation sequencing (NGS) in prenatal, postnatal diagnosis, and screening of hemoglobinopathies described in the literature and to discuss the diagnostic utility of NGS in the most frequent recessive Mendelian inherited monogenic disease worldwide, the hemoglobinopathies (Williams and Weatherall, 2012).

### The Role of NGS in the Molecular Screening of Thalassemia Carriers

Large-scale premarital carrier screening for alpha- and or beta-thalassemia has been described in the Chinese population using NGS. Preliminary data have shown that NGS may be more accurate as a first-tier DNA screening tool than conventional thalassemia screening. In addition to the higher sensitivity of carrier detection, it has also led to the identification of new variants.

He et al. (2017) used a targeted NGS approach covering the globin gene cluster for a large-scale population carrier screening program among 951 individuals of the Dai population in Yunnan. In a double-blind comparative study, the authors detected a thalassemia carrier rate of 49.5% using the direct NGS screening vs. 22% using the traditional approach, including red cell indexes combined with hemoglobin electrophoresis and subsequent DNA sequencing. Almost 74.8% of alpha-thalassemia carriers and 30.5% of combined alpha- and beta-thalassemia carriers were missed in screening by the traditional approach (He et al., 2017) due to normal or borderline values of MCV, MCH, and HbA_2_ typically found in the genotypes (-α^3.7^/α α,- α ^4.2^/α α, α ^CS^ α/α α, α ^WS^ α/α α). Not all of these minor variants of the alpha-globin genes play a role in the prevention of Hb Bart’s hydrops fetalis, while on the other hand, it contributes to the increased risk for HbH disease in the offspring of couples with one partner being alpha 0-thalassemia carrier.

A large study by Shang et al. (2017) including 10,111 couples demonstrated that NGS-based screening analysis covering the globin gene cluster and four modifying genes (KLF1, BCL11A, HBS1L, and MYB) identified 4,840 mutant alleles in 4,180 individuals. In total, 186 couples at risk of having affected offspring were identified, 35/186 of which would have been missed by traditional diagnostic screening. In addition, 12.1% of variants identified by the described NGS assay, would have remained undetected by the conventional methods involving selective potential carriers based on hematology and fraction identification methods, such as HPLC or CE, followed by Gap-PCR, MLPA, reverse dot blot (RDB), and Sanger sequencing (Shang et al., 2017). Preliminary studies by PCR-NGS among 57,229 cases were performed in Guangxi, China, and revealed uncommon or novel mutations (458 mutations in total) that could not be detected by conventional methods (Munkongdee et al., 2020).

Another approach was the combined NGS and Gap-PCR screening aiming to detect the common deletions responsible for 80% of the molecular causes of alpha-thalassemia, which are not routinely identified with short-read sequencing platforms. In this study, amongst 15,807 samples, 1,704 thalassemia carriers (prevalence 10.8%) were detected using a combination of hematology assays, Gap PCR, and NGS analysis. The prevalence rates of alpha-thalassemia, beta-thalassemia, and combined alpha- and beta-thalassemia were 5.97% (943/15,807), 4.48% (708/15,807), and 0.34% (53/15,807), respectively. Combined NGS and Gap-PCR have detected 40 genomic variants, including 11 rare and novel ones. Among these variants, traditionally combined RDB and Gap-PCR could detect only three deletions and 20 types of mutations. In addition, four novel thalassemia mutations and one novel abnormal hemoglobin mutation were identified by the combined NGS-PCR approach (Zhang et al., 2019). Besides, Zhao et al. (2020) have compared the combined gap-PCR and NGS method, to the routine workflow (red cell indexes, hemoglobin electrophoresis, followed by Gap PCR, and/or DNA sequencing) among 944 couples pre-pregnancy. The hematology and biochemical assays showed a lower sensitivity of 61% and a higher missed diagnosis ratio of 39% for alpha-thalassemia mutations (Zhao et al., 2020). Thus, these two studies indicate that combined hematology, GAP PCR and NGS, is a cost-effective approach to screen for thalassemia on a large scale.

### The Role of NGS in Molecular Diagnosis

Sanger sequencing of the amplified globin gene fragments has always traditionally been the golden standard in routine molecular diagnosis of thalassemia and Hb variants, because of the small size of the alpha- and beta-globin genes (approx. 1,200 bp and 1,800 bp, respectively). When an indication of a possible hemoglobinopathy was found in the family history, microcytic hypochromic parameters or abnormal separation on IEF, HPLC, CE, or Sanger sequencing was applied to detect variants in the alpha- and beta-globin genes; however, six cases of rare anemia disorders were reported, which were diagnosed by NGS as the first-tier method (Bharadwaj et al., 2020; Rizzuto et al., 2021). Globin gene abnormalities were not expected since the results from the biochemical analysis were normal and there was no indication in the family history. Because of the clinical suspicion of hemolytic anemia, a targeted gene panel, including the globin genes or trio WES, was performed. This identified rare unstable hemoglobin variants causing severe hemolytic anemias which were missed by biochemical assays (Bharadwaj et al., 2020; Rizzuto et al., 2021). Five of the reported rare unstable hemoglobin variants were related to the beta-globin genes: Hb Köln (Bharadwaj et al., 2020), Hb Bristol, Alesha, Hb Debrousse, Hb Zunyi, and finally, a novel elongating Hb variant called Hb Mokum. Only one case was caused by a mutation in the alpha-globin gene leading to Hb Evans (Rizzuto et al., 2021). Three out of six DNA variants were *de novo*, which explained why the parents were normal and an inherited trait was not expected.

Whole exome sequencing has also led to the identification of a new *trans-*acting candidate in beta-thalassemia, acting as a genocopy. Several members of two unrelated Dutch families showing beta-thalassemia trait with a characteristic of elevated HbA_2_ and microcytic hypochromic anemia were analyzed by Sanger sequencing, which revealed two completely normal copies of the HBB gene. WES uncovered two different pathogenic splice site variants in the *SUPT*5*H* gene (ENSG00000196235.14) that encodes the Spt5H protein, a component of the DSIF complex. A total of eight different pathogenic variants in the *SUPT*5*H* gene have been identified in 25 patients with a similar beta-thalassemia minor phenotype showing no abnormalities in the HBB gene (16, Dutch; 2, French; and 7, Greek) (Achour et al., 2020).

Finally, NGS has been used to establish the genotype- and phenotype-correlation of the alpha-thalassemia X-linked intellectual disability (ATR-X) and the ATR-16 syndrome. WES of two boys with white matter changes showed an association with ATR-X as reported by Lee et al. (2015). Likewise, Babbs et al. (2020) have reported three family members with ATR-16 syndrome presenting alpha-thalassemia, intellectual disability, developmental delay, speech delay, and facial dysmorphism. These severe phenotypes are generally caused by deletions >1 Mb contrasting with the 967 kb deletion identified in the siblings. Whole genome sequencing (WGS) was performed on specimens from the three siblings and identified a shared non-sense variant in the SMD6 gene (chromosome 15), a negative regulator of the bone morphogenetic protein signaling pathway reported to underlie craniosynostosis, speech delay, global developmental delay, fine motor impairment, and aortic valve abnormalities with variable penetrance (Babbs et al., 2020).

The identification of copy number variation and characterization of deletion/duplication breakpoints in molecular diagnostics for hemoglobinopathies is routinely done using traditional methods, such as gap-PCR and MLPA.

Recently, Rangan et al. (2019) have compared long-range read sequencing methods to standard methods to identify causative mutations in complex thalassemia cases involving the beta-globin gene cluster. This showed impressive superiority and comprehensiveness to Sanger sequencing, MLPA, array CGH, and short-range sequencing technology. First, single nucleotide variants (SNVs) have been identified with a sensitivity and specificity of 99.5%. Then, large structural variants (SVs), such as large deletions, duplications, insertions, crossovers, and fusions spanning many kilobases, were characterized in the heterozygous, homozygous, and compound heterozygous state to a precise genomic coordinate. Finally, phasing SNVs identified Hb S haplotypes (Rangan et al., 2019).

### The Role of NGS in Non-invasive Prenatal Diagnosis (NIPD) and Preimplantation Diagnosis (PGD)

With the advent of massive parallel sequencing (MPS), the sensitivity and precision of NIPD) on free fetal DNA in maternal circulation has been greatly enhanced. Hence, many researchers have developed new approaches based on NGS to apply NIPD to monogenic diseases, and beta-thalassemia was one of the models intensively studied. The first approach was based on the relative haplotype dosage analysis (RHDO), either by shotgun genome-wide sequencing (Lo et al., 2010) or by targeted sequencing (Lam et al., 2012) of genomic regions of interest. The principle of RHDO is to deduce the fetal inheritance of maternally transmitted mutations by quantifying the relative dosage of haplotypes looking at single nucleotide polymorphisms (SNP) in and around the target gene. These two studies clearly showed both clinical feasibility and utility of NIPD in beta-thalassemia based on PCR methodologies and NGS strategies (Lo et al., 2010; Lam et al., 2012). Vermeulen et al. (2017) utilized target locus amplification (TLA) in NIPD to achieve robust haplotyping in parents of affected offspring without the need to analyze other first-degree family members. TLA involves an initial step to crosslink physically the proximal sequences in the parental DNA. The subsequent sequencing of the targeted crosslinked region (e.g., the *HBB* gene cluster) using just a few primers facilitates sequencing of 10–100 kb across the locus of interest and the subsequent derivation and phasing of parental haplotypes. Targeted deep sequencing of the phased variants in cfDNA from the pregnant mother and tailored statistical analysis have allowed robust prediction of the fetal genotype relative to the disorder under investigation (Vermeulen et al., 2017). Recently, Yang et al. (2019) have developed a novel approach termed cfBEST for NIPD of monogenic disorders. Based on NGS methodology, the authors aimed to directly deduce the fetal and maternal genotypes by counting single allelic molecules and calculating the mutation ratio in cfDNA of maternal circulation without prior knowledge of parental genotypes. This approach was validated with a blinded assay among 143 pregnant women at risk for beta-thalassemia, which revealed an allele detection sensitivity of 99.1% and a specificity of 99.9% (Yang et al., 2019).

Finally, Kubikova et al. (2018) described a novel preimplantation genetic testing protocol, based on NGS technology, for the virtual detection of all mutations in the *HBB* gene. In this study, a multiplex PCR protocol has been designed allowing simultaneous amplification of multiple overlapping DNA fragments encompassing the entire *HBB* gene sequence in addition to 17 well-characterized closely linked SNP. Amplicons were subsequently analyzed using an NGS method revealing both disease-causing mutations and SNP genotypes. The *HBB* mutation status and associated SNP haplotypes were successfully determined in all 21 embryos suggesting that the combination of trophectoderm biopsy and highly sensitive NGS may provide superior accuracy than typically achieved using traditional PGD approaches (Kubikova et al., 2018).

## Discussion

Over the past few years, the scientific literature has proven the efficiency of NGS in research, diagnosis, and screening for many Mendelian inherited diseases. Currently, target gene panels and WES approaches have become methods of choice to detect mutations in many heterogeneous genetic diseases and have been adopted in many clinical laboratories. Although many studies have focused on the role of MPS in Mendelian diseases (Jennings et al., 2017; Kalayinia et al., 2018; Pipis et al., 2019; Pecoraro et al., 2020), the diagnostic application of NGS in hemoglobinopathies is still not widely adopted. The cost of NGS and the small size of globin genes, which facilitates Sanger sequencing, might have contributed to this delay; however, due to the continuous reduction in the cost of NGS sequencing, this might change in the future.

Next-generation sequencing has been studied as a tool for large-scale carrier screening of thalassemia among different populations living in China. Although all of these studies suggested the high accuracy of NGS in detecting carriers, this is still not easily reproducible in other populations and cost remains high, especially, for endemic low-income countries. In addition, several mutations revealed in these large-scale screening studies do not have clinical implications which make the utility of its detection questionable.

Similarly, hemoglobinopathies are unique in comparison with other diseases in that detection of carriers is possible using hematological and biochemical tests. Thus, in the majority of the cases, low-cost hematological and biochemical assays are still preferred, especially, in endemic countries. At the same time, mutations found by NGS need phenotypic information to be interpreted properly. The hematological and biochemical analyses represent the phenotype and therefore cannot be skipped according to best-practice recommendations. Furthermore, Gap-PCR as a screening step will remain mandatory to detect deletions that cause over 80% of alpha-thalassemias (Harteveld and Higgs, 2010). In addition, it is important to mention the technical difficulties in sequencing the globin genes due to the high degree of homology with duplicated- and pseudo-genes. Indeed, the presence of high homology between HBA1 and HBA2, HBB and HBD, and HBG1 and HBG2, and the presence of Alu repeats in the alpha-gene cluster, and LINE repeats in the beta-gene cluster interfere with the specificity of NGS. However, the detection of deletions/duplications/inversions and translocations using shorthead sequencing is still challenging (Yamamoto et al., 2016). Primarily, at present time, no data on detecting CNV in hemoglobinopathies using NGS are available. Furthermore, NGS is still more expensive than conventional DNA techniques used for hemoglobinopathies screening and diagnosis. We expect that with the improvement of either long-read NGS technologies, the introduction of WGS in the clinical diagnostic setting, the improvement of copy number variant detection and decrease in costs, MPS would find a vast place in routine laboratory diagnostics of hemoglobinopathies.

On the other hand, NGS has proven to be an efficient tool in resolving complex cases of thalassemia, congenital severe anemia, and hemolytic anemia that otherwise would have remained undiagnosed. The unstable dominant hemoglobin variants, such as reported by Bharadwaj et al. (2020) and Rizzuto et al. (2021), demonstrated the importance to include both globin genes and red cell membrane genes in gene panels to diagnose congenital anemia disorders. In the example of *SUPT5H*, NGS has allowed the identification of a new *trans-*acting factor gene involved in regulating beta-globin gene expression, which results in beta-thalassemia trait when haploinsufficient. This finding did not only contribute to the diagnosis and genetic counseling for families showing atypical beta-thalassemia intermedia but also opened new opportunities for a better understanding of the erythroid-specific beta-gene regulation of expression and possibly to future perspectives of gene therapy. NGS has also contributed for a better establishment of genotype-phenotype correlation of the hemoglobinopathies by identifying modifier genes in ATR-16. Although these investigations may not have direct clinical utility in prognosis and patient management at present, they may contribute to a better understanding of the pathophysiology of the hemoglobinopathies.

Finally, during the development of NIPD for hemoglobinopathies, many of the methods employed have been based on NGS and a common feature of these studies is the necessity to detect specific paternally inherited alleles. All these studies have in common the necessity to detect specific paternally inherited alleles. Although most of these studies demonstrated good specificity and sensitivity, they have inherent limitations, such as complicated procedures, a lack of versatility, and the need for prior knowledge of parental genotypes or haplotypes. NGS-based methodology of cfBEST has been used to directly deduce the fetal and maternal genotypes by counting single allelic molecules and calculating the mutation ratio in cfDNA of maternal circulation without prior knowledge of the parental genotypes. This method and TLA may provide a potentially practical, robust, and affordable approach for NIPD.

In conclusion, we speculate that NGS-based technology is not likely to replace existing methods but can be a useful additional tool in the diagnostic strategy of hemoglobinopathies, especially for large-scale genetic screening and in the discovery of novel causes of thalassemia. Although the costs are still high, the vast majority of cases can be solved by traditional Sanger sequencing of the relatively small HBA1, HBA2, and HBB genes, while NGS analysis cannot be sufficient to diagnose the hemoglobinopathies without using traditional methods involving hematology and Hb-typing to establish a proper genotype-phenotype correlation. On the other hand, NGS could have a vast place in the diagnosis of unresolved complex cases involving factors outside the alpha- and beta-globin gene clusters and in prenatal screening. The validation of long-range sequencing to adequately characterize deletions and duplications involving the alpha- and beta-globin genes and the decrease in costs would open future opportunities for NGS in the diagnosis of the hemoglobinopathies.

## Author Contributions

All authors listed have made a substantial, direct and intellectual contribution to the work, and approved it for publication.

## Conflict of Interest

The authors declare that the research was conducted in the absence of any commercial or financial relationships that could be construed as a potential conflict of interest.

## Publisher’s Note

All claims expressed in this article are solely those of the authors and do not necessarily represent those of their affiliated organizations, or those of the publisher, the editors and the reviewers. Any product that may be evaluated in this article, or claim that may be made by its manufacturer, is not guaranteed or endorsed by the publisher.
